# Does age alone negatively predict the outcome of sacral neuromodulation? A single-centre retrospective observational study

**DOI:** 10.1186/s12894-020-00621-6

**Published:** 2020-05-14

**Authors:** Sandra Schönburg, Tobias Bukethal, Paolo Fornara

**Affiliations:** 1 Department of Urology and Kidney Transplantation, Martin Luther University, Ernst-Grube-Straße 40, 06120 Halle (Saale), Germany; 2 Urological practice of Ralf Eckert (M.D.), Klosterstraße 2, 06295 Lutherstadt Eisleben, Germany

**Keywords:** Sacral neuromodulation, Elderly, Geriatric assessment, Outcome, Complications

## Abstract

**Background:**

For patients over the age of 70 years, sacral neuromodulation (SNM) is often not considered a potential therapeutic option. We therefore report on our results from performing SNM in elderly patients ≥70 years.

**Methods:**

Between 01/09 and 12/18, a total of 95 patients with refractory overactive bladder (OAB) or chronic non-obstructive urinary retention underwent SNM testing at our department. In the overall sample, 20 patients were aged 70 years or older (21%, group B), and 75 patients were under 70 years old (79%, group A). The mean follow-up period was 50.2 ± 36.2 months. Pre-, peri- and postoperative parameters were compared between the two groups. Statistical analysis was carried out with SPSS 25.0 (*p* < 0.05).

**Results:**

The mean patient age was 53 ± 16 (17–76) years. The indications for SNM testing were OAB and retention in 51 and 49% of patients, respectively. A total of 56 patients (59%) [8 patients (40%) in group B, 48 patients (64%) in group A] had more than 50% improvement in the context of the test (stage 1), such that a permanent neuromodulator (stage 2) was implanted. A total of 14 patients, all under 70 years old except for one older female, needed to undergo revision due to defects or infection. Overall, the success rate was 58.3% for OAB and 59.6% for urinary retention. The success rates and complications in our patient group were independent of age and geriatric assessment.

**Conclusion:**

SNM can also be successfully implemented in older patients.

## Background

The average age of the European population is increasing, as is the age of our patients with bladder dysfunction [1–3]. We live in times of demographic change [4], in which we must pay special attention to our elderly patients. Multimorbidity, frailty with frequent falls, and cognitive and functional restrictions, especially in connection with polypharmacy, are important factors that need to be considered [5–7]. However, is it possible to predict the success or failure of a treatment based on the patient’s chronological age? Especially for highly specialized interventions, such as sacral neuromodulation (SNM), it is important to consider to what extent the patient’s age has implications for therapeutic success or possible complications. The best-practice statement of the International Continence Society (ICS) for the use of SNM [8] makes no direct recommendation but states that any cognitive impairment rendering the patient unable to manage the device is an absolute contraindication for SNM. In daily practice, SNM is often not considered as a potential therapeutic option for patients aged 70 years or older. However, the literature contains only a few, sometimes very differently structured studies addressing this question [9–12], and these studies report partially conflicting results concerning the relationship between patient age and the outcome of SNM. In those studies, only chronological patient age was considered. There was no further differentiation using a geriatric assessment, for example. The present study therefore examines the therapeutic outcome as well as the complications and revisions of SNM in elderly patients differentiated according to age and geriatric assessment.

## Methods

### Study design

In the present single-centre retrospective observational study (Halle (Saale), Germany), we evaluated data from 95 patients who underwent SNM testing in our urological department between January 2009 and December 2018. The approval of the local ethics committee and the consent of the patients were obtained for the examination. All patients were supervised during an individual bladder dysfunction consultation in an outpatient clinic.

### Patient population

The prerequisites for SNM testing included bladder disorders such as refractory overactive bladder (OAB, with or without urge incontinence) or chronic non-obstructive urinary retention lasting at least 6 months as well as failure of conservative treatments (e.g., lifestyle modifications, pelvic floor exercises, biofeedback, anticholinergic or cholinergic medication and (self-)catheterization). Untreated urethral stricture, an existing or planned pregnancy or diagnosed intellectual disability was considered a contraindication for SNM testing. Patients with any of these contraindications received other therapies and did not undergo SNM; therefore, those patients were not included in the evaluation of the present study. A geriatric assessment, the ISAR (Identification of Seniors at Risk) score [13], was routinely administered to each patient on admission to the hospital. For this study, the ISAR score was extracted from the admission information for SNM testing (stage 1) and included in the analysis. In addition, the CCI (Charlson Comorbidity Index) and the ASA score (American Society of Anaesthesiologists risk classification) were also determined and included in the analysis [14–16].

### Data collection

The surgical procedures were performed routinely as previously described [17, 18]. The definition of “success” was as follows. For stage I, success was considered if the patient experienced more than 50% improvement in one or more of the bothersome parameters [8] (urinary frequency, incontinence episodes or voided volume) during the four-week stage I period. Furthermore, the patients were retrospectively reviewed from stage I lead placement until their last clinic follow-up visit by December 2019 to achieve a follow-up of at least 12 months. The mean follow-up period of the study was 50.2 ± 36.2 months. Thus, the follow-up endpoint for the present study was December 2019. For the follow-up period, success was considered if the patient continuously experienced more than 50% improvement in the respective bothersome parameter [8] without any other therapy for bladder dysfunction. During the follow-up period, patients were evaluated for continuous improvements, postoperative complications and device removal. Postoperative complications were defined according to the Clavien-Dindo classification [19, 20]. Battery replacement was not considered a postoperative complication.

### Statistical analysis

The primary outcome of the present study was the efficacy of SNM, which was determined by the rate of progression to stage II. Secondary analyses evaluated postoperative complications and device removal. Other variables analysed in the present study included demographic data, geriatric assessment results, comorbidities, diabetes mellitus, prior OAB and retention therapy and battery replacement as a regular indication for revision surgery.

Statistical analysis was performed with SPSS for Windows (IBM Corp., released 2017; IBM SPSS Statistics for Windows, Version 25.0. Armonk, NY: IBM Corp.). Data are presented as the mean ± standard deviation (SD) and range. Statistical significance was defined as *p* < 0.05. The bivariate comparisons were conducted with unpaired Student’s t-tests (numerical variables) or chi-squared tests (categorical variables).

### Statistical limitations

The present study is a retrospective descriptive investigation with inherent statistical limitations, e.g., the patient groups differ in size (group A: 75 patients, group B: 20 patients) and unpaired Student’s t-tests (numerical variables) or chi-squared tests (categorical variables) could be used for the statistical bivariate comparisons only if a minimum number of values was available for the respective category. If the minimum number was not reached, “lack of items” was documented.

## Results

### Baseline characteristics

The baseline characteristics of the overall sample are shown in Table 1. On average, the patients were 53.2 ± 16.1 (17–76) years old, slightly overweight [body mass index (BMI): 27.5 ± 5.4 (16.2–43.2) kg/m^2^] and slightly comorbid [CCI: 0.47 ± 0.766 (0–3)], including diabetes mellitus in 9.4% of cases. With regard to the indication for SNM, our overall sample was balanced. Forty-eight patients (51%) and 47 patients (49%) suffered from refractory OAB and retention problems, respectively. In terms of gender, women made up a slight majority, with a share of 62%. Of the overall population, 20 patients were aged 70 years or older. The oldest patient was 76 years old. In the comparison of the two age groups considered (group A, patients < 70 years old, versus group B, patients ≥70 years old), OAB rather than retention disorder tended to be more common in older patients (group A: OAB 47%, group B: OAB 65%, *p* = 0.145). As measured by the CCI, older and younger patients had similar degrees of comorbidity (*p* = 0.620). The proportion of patients with diabetes mellitus also did not differ at approximately 10% in each group. No difference was found between the age groups with regard to the anaesthesiology assessment using ASA scores (group A: ASA 1, 7%, ASA 2, 68%, ASA 3, 25%; group B: ASA 1, 5%, ASA 2, 65%, ASA 3, 30%). With regard to the geriatric assessment, an ISAR score of 3 was more frequent in the older patients (group B, 10%) than in the younger patients as expected (group A, 3%; Table 1, Additional file 1).

**Table 1:** Baseline characteristics of the patient population

Category	Overall population *n* = 95 pts	A: Patients < 70 years *n* = 75 pts	B: Patients ≥70 years *n* = 20 pts	*P*-value “patients < 70 years” versus “patients ≥70 years”
**Patient age [years] (range)**	53.2 ± 16.1 (17–76)	48 ± 13.6 (17–69)	73 ± 2.0 (70–76)	–
**Gender (%):**				
**• Male (%)**	36 (38)	29 (39)	7 (35)	0.764*
**• Female (%)**	59 (62)	46 (61)	13 (65)	
**BMI [kg/m**^**2**^**] (range)**	27.5 ± 5.4 (16.2–43.2)	27.5 ± 5.6 (16.2–43.2)	27.3 ± 4.4 (19.3–36.2)	0.872**
**ASA score (%):**				
**• ASA 1 (%)**	6 (6)	5 (7)	1 (5)	Lack of items.
**• ASA 2 (%)**	64 (67)	51 (68)	13 (65)	
**• ASA 3 (%)**	25 (26)	19 (25)	6 (30)	
**CCI score (range)**	0.47 ± 0.766 (0–3)	0.45 ± 0.776 (0–3)	0.55 ± 0.759 (0–3)	0.620**
**Diabetes mellitus (%)**	9 (9.4)	7 (9.3)	2 (10)	Lack of items.
**ISAR score (%):**				
**• ISAR score 0**	45 (47)	39 (52)	6 (30)	Lack of items.
**• ISAR score 1**	38 (40)	28 (37)	10 (50)	
**• ISAR score 2**	8 (8)	6 (8)	2 (10)	
**• ISAR score 3**	4 (4)	2 (3)	2 (10)	
**Cause of bladder function disorder (%):**				
**• OAB (%)**	48 (51)	35 (47)	13 (65)	0.145*
**• Retention (%)**	47 (49)	40 (53)	7 (35)	
**OAB classification (%):**				
**• OAB dry (%)**	16 (33)	11 (31)	5 (39)	0.645*
**• OAB wet (%)**	32 (67)	24 (69)	8 (61)	
**Prior OAB therapy (%):**				
**• Oral medication (%)**	48 (48/48, 100)	35 (35/35, 100)	13 (13/13, 100)	0.249*^#^
**• Onabotulinumtoxin A (%)**	23 (23/48, 48)	15 (15/35, 43)	8 (8/13, 62)	
**Prior retention therapy (%):**				
**Oral medication (%)**	8 (8/47, 17)	8 (8/40, 20)	0	Lack of items.
**Catheterization (%)**	39 (39/47, 83)	32 (32/40, 80)	7 (7/7, 100)	

(*n* = 95 patients); the mean ± standard deviation (range) or percentage

*Chi-square test (α = 0.05), **T-test unpaired (α = 0.05), ^#^Onabotulinumtoxin vs. no onabotulinumtoxin

### Peri- and postoperative parameters

The peri- and postoperative parameters of the overall sample are listed in Table 2. With regard to the operative time, SNM testing (stage 1) took an average of 32 ± 18 (6–81) minutes, and device implantation (stage 2) took slightly more than 1 h (62 ± 24 (17–140) minutes). The patients were hospitalized for device implantation (stage 2) for an average of 5.0 ± 1.4 (3–8) days; in general, the duration of hospitalization must be considered in accordance with the German health care system. In the comparison between age groups, there was no statistically significant difference in operative time or hospitalization time (operative time: stage 1 *p* = 0.484, stage 2 *p* = 0.264; hospitalization: *p* = 0.619). Regarding the success of stage 1, older patients tended to have worse outcomes (64% success in the younger patients (*n* = 48) and only 40% in the older patients (*n* = 8)), but this difference was, once again, not statistically significant (*p* = 0.053). Overall, SNM testing had a positive outcome in 56 (59%) patients, which is a very good result for third-line therapy. Interestingly, almost all candidates with an ISAR score of 3 met the criteria to receive the SNM device (Fig. 2).

**Table 2:** Peri- and postoperative parameters of the patient population

Category	Overall population *n* = 95 pts	A: Patients < 70 years *n* = 75 pts	B: Patients ≥70 years n = 20 pts	P-value “patients < 70 years” versus “patients ≥70 years”
**Stage 1 (%)**	95	75	20	–
**Successful stage 1 (%)**	56 (59)	48 (64)	8 (40)	0.053**
**Underwent stage 2 (%)**	56 (59)	48 (64)	8 (40)	0.053**
**Operative time stage 1 [min] (range)**	32 ± 18 (6–81)	31 ± 18 (6–81)	34 ± 17 (11–77)	0.484**
**Operative time stage 2 [min] (range)**	62 ± 24 (17–140)	63 ± 24 (17–140)	53 ± 21 (30–88)	0.264**
**Hospitalization**^a^**stage 2 [d] (range)**	5.0 ± 1.4 (3–8)	5.0 ± 1.4 (3–8)	4.75 ± 1.6 (3–8)	0.619**

(*n* = 95 patients); the mean ± standard deviation (range) or percentage

**T-test unpaired (α = 0.05)

^a^Hospitalization in accordance with the German health care system

### Postoperative complications and revisions – stage 1

As expected, no intraoperative complications were observed. Postoperative complications regarding stage 1 (Table 3) were rare and included only two adverse events. One patient had a urinary tract infection (Clavien I), which was treated with an antibiotic selected according to a microbiological examination. In another patient, a wound infection was found in the area of the test electrodes, necessitating early removal of the electrodes (Clavien IIIb). In this case, the test was assessed as negative and was not repeated at the patient’s request. Both adverse events were observed in the younger age group. Due to the rarity of both events, no statistical significance could be detected.

**Table 3:** Postoperative complications among the patient population during stage 1

Clavien grade – Postoperative complications^a^ stage 1		Overall population n = 95 pts	A: Patients < 70 years *n* = 75 pts	B: Patients ≥70 years *n* = 20 pts	*P*-value “patients < 70 years” versus “patients ≥70 years”
**Clavien I**	Urinary infection	1 (1.1)	1 (1.3)	0	Lack of items.
**Clavien IIIb**	Wound infection stage 1 (< 30 days)	1 (1.1)	1 (1.3)	0	
**Sum total**		**2 (2/95, 2.1%)**	**2 (2/75, 2.7%)**	**0**	

(*n* = 95 patients), 2 postoperative complications in 2 patients

^a^Postoperative complications according to the Clavien-Dindo classification [19, 20], presented as the number and percentage

### Postoperative complications and revisions – stage 2

No intraoperative complications were observed at stage 2. Postoperative complications regarding stage 2 are shown in Table 4. A total of 18 complications occurred in 17 patients (18/56, 32.1%); specifically, 3 patients had slight complications of Clavien grade I (3/56, 5.4%), and 14 patients had Clavien grade IIIb complications that necessitated revision (15/56, 26.8%).

**Table 4:** Postoperative complications among the patient population during stage 2

Clavien grade – Postoperative complications^a^ stage 2		Overall population *n* = 56 pts	A: Patients < 70 years *n* = 48 pts	B: Patients ≥70 years *n* = 8 pts	P-value “patients < 70 years” versus “patients ≥70 years”
**Clavien I**	Urinary infection	1 (1.8)	1 (2.1)	0	Lack of items.
	Haematoma	1 (1.8)	1 (2.1)	0	
	Pain	1 (1.8)	1 (2.1)	0	
**Clavien IIIb**	Pain necessitating device removal	0	0	0	
	Wound infection at implantation site (< 30 days)	0	0	0	
	Wound infection at implantation site (> 30 days)	1 (1.8)	1 (2.1)	0	
	Erosion and infection at implantation site (> 30 days)	3 (5.4)	3 (6.3)	0	
	Subacute erosion at implantation site (> 30 days)	1 (1.8)	1 (2.1)	0	
	Lead fracture or migration (> 30 days)	6 (10.7)	5 (10.4)	1 (12.5)	
	Urgently indicated MRI with necessity for device removal	1 (1.8)	1 (2.1)	0	
	Loss of efficacy necessitating device removal	3 (5.4)	3 (6.3)	0	
**Sum total**		**18 (18/56, 32.1%)**	**17 (17/48, 35.4%)**	**1 (1/8, 12.5%)**	

(*n* = 56 patients), 18 postoperative complications in 17 patients

^a^Postoperative complications according to the Clavien-Dindo classification [19, 20], presented as the number and percentage

Regarding the Clavien I complications, there was a patient with a urinary tract infection that needed antibiotic treatment; a patient with a haematoma near the implantation site, which showed spontaneous and gradual resolution with local cooling and local wound management; and a patient with prolonged postoperative pain in the area of the implantation site (initial visual analogue scale (VAS) postoperatively: 6/10), which also completely resolved after a four-week analgesic regimen of nonsteroidal anti-inflammatory drugs (NSAIDs). None of the Clavien I complications had any further therapeutic consequences. All Clavien I complications affected patients < 70 years of age. Regarding Clavien grade IIIb complications, 3 patients (5.4%) had cutaneous erosion from the foreign material and subsequently developed wound infection, requiring surgical revision and removal of the entire device; additionally, 6 patients (10.7%) developed fracture or migration of the electrodes and needed surgical revision and new electrodes or position correction. All other observed Clavien grade IIIb complications were rare; the specifics of these complications were as follows. A kidney transplant recipient had an initially unnoticed, subclinical wound infection at the implantation site and subsequently needed the entire device removed. Furthermore, a very lean patient (BMI: 16.2 kg/m^2^) developed subacute cutaneous erosion from the device and fractured one electrode due to a fall. Finally, a young patient suffered rapid progression of his underlying neurological disease and urgently needed to undergo MRI, which necessitated the removal of the SNM device. In a total of 5 patients (8.9%), the device needed to be removed. Only one patient was re-implanted successfully. Upon individual request, the other 4 patients were not re-implanted. The loss of the effect of stimulation was also considered a postoperative complication and was found in only 3 younger patients (5.4%). As a result, all postoperative complications of stage 2, except for one electrode fracture in an older female due to a fall on the buttocks, occurred in the younger age group.

### Battery replacement

A weak battery that needed to be replaced (Table 5) was not considered a postoperative complication, since it is not an undesirable event but a natural consequence of using the SNM device. Battery replacement was required in a total of 4 patients (4.2%), comprising 3 patients under 70 years old and one patient over 70 years old. In this regard, no statistical significance was given.

**Table 5:** Regular indications for revision surgery among the patient population

Regular indications for revision surgery	Overall population *n* = 56 pts	A: Patients < 70 years *n* = 48 pts	B: Patients ≥70 years *n* = 8 pts	P-value “patients < 70 years” versus “patients ≥70 years”
Low battery necessitating change (%)	4 (4.2)	3 (4)	1 (5)	Lack of items.

(*n* = 56 patients), 4 battery changes in 4 patients, presented as the number and percentage

### Statistical analysis

**Fig. 1:**
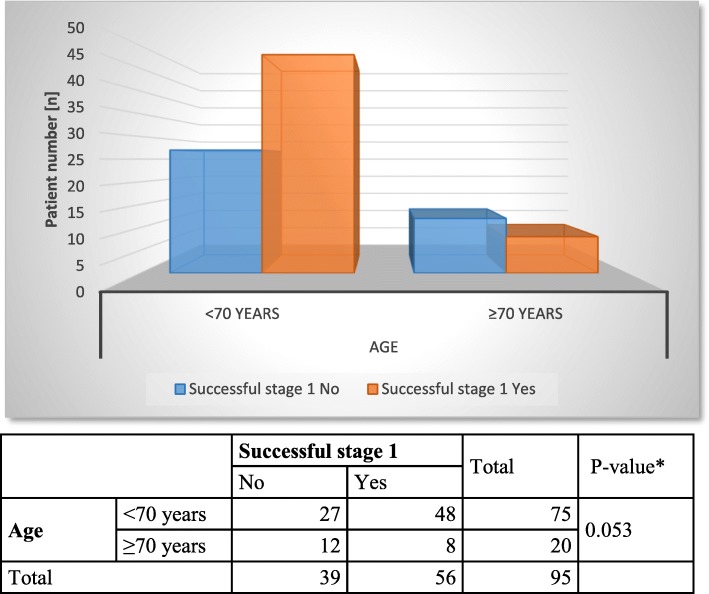
Comparison of age and success for stage 1

**Fig. 2:**
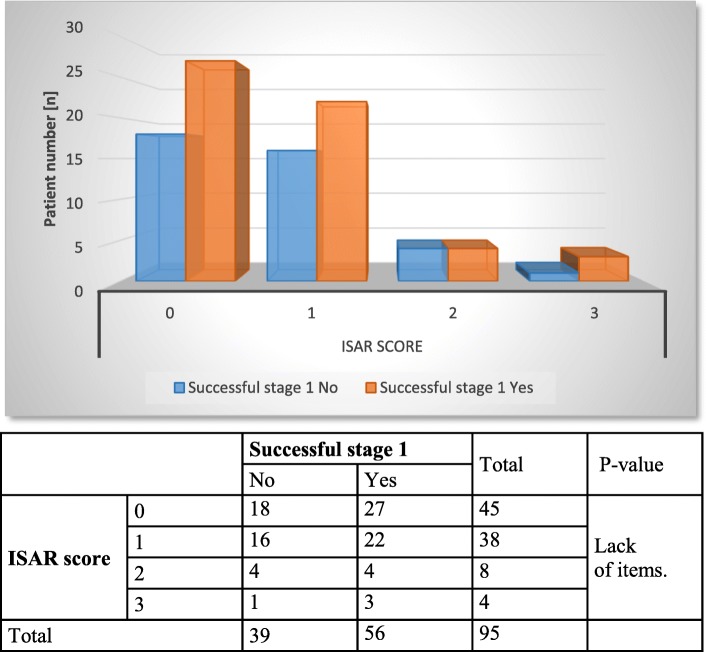
Comparison of ISAR scores and success for stage 1

**Fig. 3:**
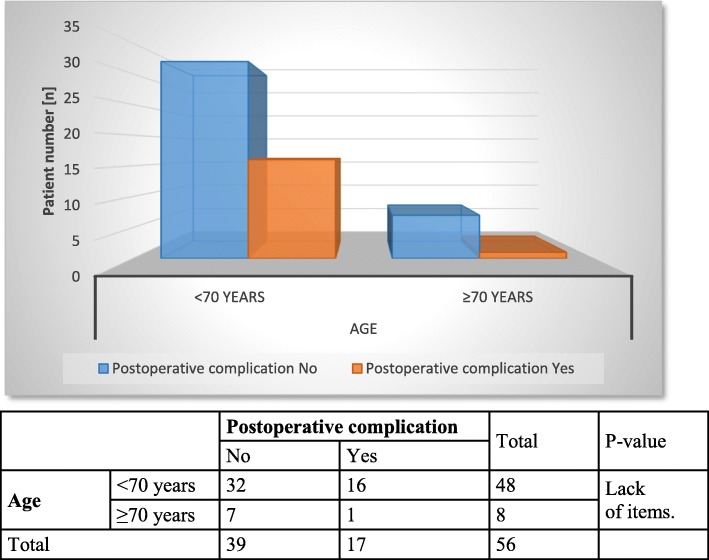
Comparison of age and postoperative complications

**Fig. 4:**
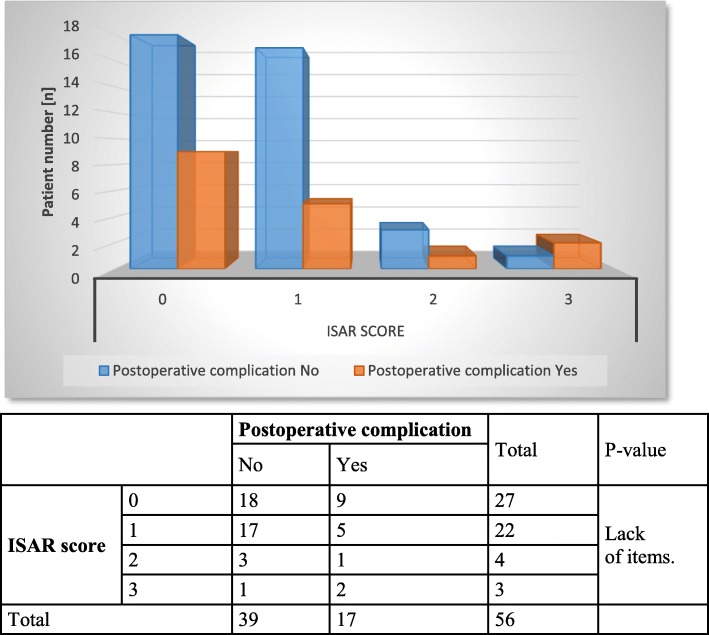
Comparison of ISAR scores and postoperative complications

**Fig. 5:**
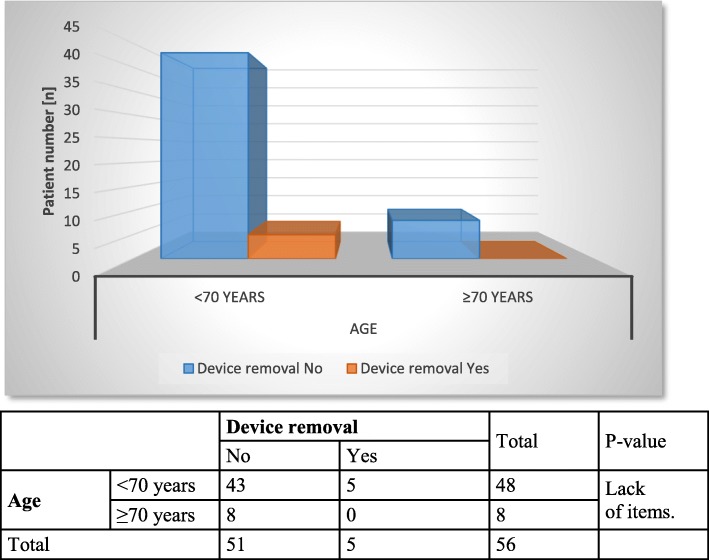
Comparison of age and device removal

**Fig. 6:**
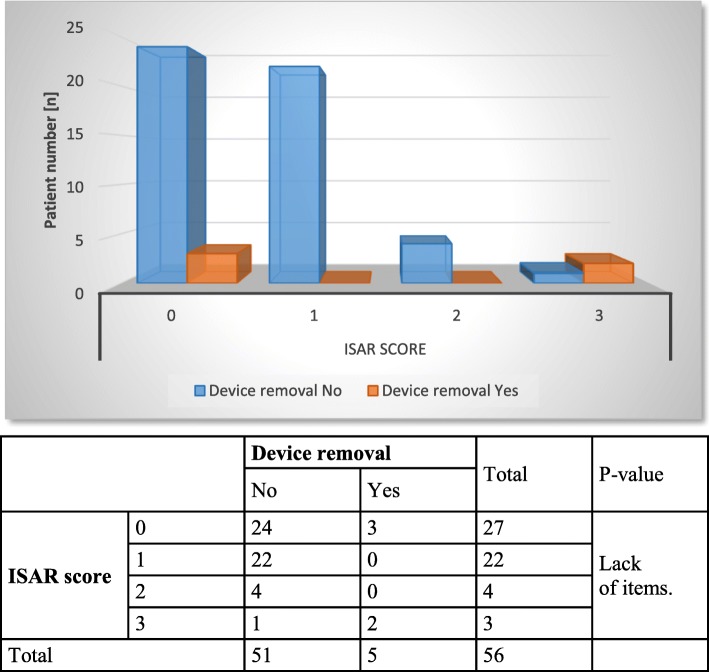
Comparison of ISAR scores and device removal

In further statistical analysis, chronological patient age and ISAR scores were correlated with the therapeutic success of stage 1 (Figs. 1 and 2), postoperative complications (Figs. 3 and 4) and device removal (Figs. 5 and 6). Regarding the patient age, older patients (aged ≥70 years) tended to have a lower therapeutic success rate for stage 1 (*p* = 0.053, Fig. 1) but fewer postoperative complications (group A: 16 patients, group B: 1 patient, Fig. 3) and device removals (group A: 5 patients, group B: 0 patients, Fig. 5). To ensure data quality with the age cut-off of “70 years” and considering the possibility that a true difference may exist for other age cut-offs, the success of stage 1, postoperative complications and device removals were also compared for age cut-offs of 65, 60 and 55 years (Additional file 2), but no significant difference in success was found for these age cut-offs. For the other parameters - postoperative complications and device removals - the statistical evaluation was restricted, but both remained low in the older patient group, with no device removal in patients older than 55 years. For the geriatric assessments, which were also restricted in terms of the statistical evaluation, the data showed that the rates of postoperative complications (ISAR score 3: 67%) and device removals (ISAR score of 3: 67%) were increased in patients with an ISAR score of 3, and all patients who needed the device removed were under 70 years of age (Fig. 5).

## Discussion

Sacral neuromodulation (SNM) is a minimally invasive therapy that activates the residual physiological function of the pelvic floor by electrically modulating the afferents of the sacral plexus, optimizing nerve communication; as a result, bladder, bowel and sexual function are normalized [21–23]. SNM has been in clinical use since the 1990s and represents a successful third-line therapy, with success rates up to 80% for many patients who are formally considered to be “out of therapy” [22]. The classic indications in urology are refractory OAB with or without urge incontinence, chronic non-obstructive urinary retention and chronic pelvic pain syndrome. On the proctological side, SNM also represents a therapy option for the treatment of faecal incontinence and functional constipation.

The ever-present demographic change in the industrialized world [4] means that an increasing number of older patients are being treated in all areas of medicine today. Geriatric medicine deals with the special diseases of elderly and multimorbid patients, typically older than 65 years [1, 13, 24]. The average geriatric patient is over 70 years old. For highly specialized operations such as SNM, it is important to consider to what extent the age of the patient has an impact on the success rate, complications and revisions. The literature contains contradictory reports on this topic [9–12]. The main barriers to generalizability are the different thresholds used to separate young and old patients. For example, Amundsen et al. [25] and Anger et al. [9] selected an age limit of 55 years as the boundary between young and old. In comparing of these two age groups, Amundsen et al. [25] found that the therapeutic success rate was significantly lower in patients over 55 years old than in younger patients, whereas Anger et al. [9] could not observe any differences. Lee et al. [10] and Greenberg et al. [12] placed the threshold at 80 years and found no age-related difference in the success rate of SNM. Faris et al. [11] divided their study population into individual decades of life and found no difference in the outcome of SNM. In the abovementioned studies, only chronological patient age was considered in the correlation analysis; no further differentiation was made according to geriatric assessment scores. How uniform is the “old” population? Do the older SNM patients belong to a “healthy old” subset of elderly patients? Further differentiation by means of geriatric assessment is helpful here; although the geriatric assessment itself is diverse and sometimes extensive, it has a valuable ability to capture different domains of functionality in ageing people [24]. The present study therefore examines the therapeutic outcomes as well as the complications and revisions of SNM in geriatric patients in the context of chronological age as well as geriatric assessment, based on a representative follow-up period (50.2 ± 36.2 months) in our own sample of SNM patients (*n* = 95).

Regarding baseline characteristics, no significant differences were found between the two age groups. An ISAR score 3 (B: 10%; A: 3%) and the presence of OAB disorders (B: 65%; A: 47%) tended to be slightly more frequent in patients ≥70 years old than in younger patients; however, neither difference was significant. With regard to the geriatric assessment, we opted to use the ISAR score [13] because it is routinely collected within our department as part of every inpatient admission. The score is a very simple geriatric assessment tool [26, 27] that is often used in everyday clinical practice because it is a questionnaire consisting of only six questions. A clinician fills out the questionnaire together with the patient or a caregiver and asks about the need for help, acute changes in the need for help, recent hospitalization, sensory and cognitive limitations, and multimorbidity as indicated by polypharmacy. The screening is considered positive if the patient scores three or more points. Geriatric assessment using the ISAR score thus reflects functionality and cognition regardless of chronological patient age. As a result, geriatric assessment using the ISAR score can also be positive in patients under 70 years old, as our data confirm (see Additional file 1). To collect further comparison parameters that might be able to differentiate patients, we also carried out an evaluation using CCI and ASA scores. The CCI estimates patient morbidity using 19 prognostically relevant secondary diseases, especially cardiovascular events, liver diseases and dementia. The CCI is a highly reliable and well-studied instrument that is quick and easy to use. According to the CCI, our total population showed only slight comorbidity (0.47 ± 0.766 (0–3)), with no significant difference between the two age groups. Consequently, our elderly patients had only a few comorbidities. In contrast, approximately 25–30% of our patients had an ASA score of 3, indicating a serious general illness with reduced performance. However, the ASA score is a somewhat subjectively influenced parameter, dependent on the anaesthesiologist’s perspective regarding the individual anaesthetic risk of the patient. Interestingly, in a comparison of the two age groups, which was also restricted in terms of the statistical evaluation, we found no difference in ASA scores (group A: ASA 1, 7%, ASA 2, 68%, ASA 3, 25%; group B: ASA 1, 5%, ASA 2, 65%, ASA 3, 30%). The older patients had an anaesthetic risk similar to that of the younger patients in our population.

With regard to the success rate of SNM, our result was similar to those of most previous studies [9–12]. There was no significant difference in the success of the procedure according to age. On closer inspection, however, therapeutic outcomes tended to be poorer in the older patients (*p* = 0.053, Fig. 1), possibly due to the small sample size. To ensure that the age cut-off of “70 years” did not mask a true difference, we compared the success of stage 1 for additional age cut-offs of 65, 60 and 55 years (see Additional file 2) but found no significant difference in the success. Regarding postoperative complications, we found that older patients tended to have fewer postoperative complications (group A: 16 patients; group B: 1 patient, Fig. 3), similar to the results of Faris et al. [11]. However, the small sample size of the elderly group may make a clean statistical analysis difficult. In a larger patient population (*n* = 356 patients), Faris et al. [11] found a reduced revision rate in elderly patients and suggested that the elderly patients might have been unable to undergo further anaesthesia safely. However, we evaluated all postoperative complications (see Tables 3 and 4) and found that fewer complications were observed in the older patients (see Fig. 3).

In our patient sample, there were only two operative revisions in patients ≥70 years: one revision due to an electrode fracture because of a fall on the buttocks in an older patient with an ISAR score of 1 and a scheduled revision to replace an exhausted battery in one older patient with an ISAR score of 0. No need for device removal was observed in the group of patients aged ≥70 years (see Fig. 5). Similar to the information in the literature (4–9%) [28–30], a total of 5 devices (8.9%) had to be removed; removal was significantly more common in patients with a positive geriatric assessment than in those with a negative assessment, but there was no effect of patient age (see Figs. 5 and 6).

In summary, our data showed that older patients can be sufficiently physically and mentally healthy for SNM. The difficulty lies in accurately distinguishing these patients. Chronological age, geriatric assessment and comorbidity scores can be helpful, but these factors alone do not ultimately decide the pros and cons of SNM. In the case of an existing indication for SNM, a firm and medically well-founded decision by the surgeon and patient depends, above all, on the surgeon’s assessment and expertise, as well as suitable advice and education for the patient regarding the chances of success and the possible complications of SNM.

The limitations of this study include the lack of an SNM control group as well as the heterogeneous patient sample, which ultimately corresponds to the demographics encountered in daily practice. Additionally, our sample was, of course, a highly selected patient group in which we attempted retrospectively to understand the individual decision-making process. A generalization to other interventions is therefore difficult or impossible due to the highly selected patients and the low invasiveness of SNM. Further investigations comparing SNM to other therapy options, if necessary, must follow.

## Conclusions

Sacral neuromodulation can be successfully implemented even in older patients. Advanced patient age alone should therefore not be a reason for exclusion. However, SNM candidates must undergo rigorous selection that, in addition to parameters such as age, geriatric assessment and analysis of comorbidities, depends above all on cognitive abilities. In contrast, our data do not suggest that an increased rate of postoperative complications, surgical revisions, or device removal is to be expected in elderly patients.

## Supplementary information

**Additional file 1.** Comparison of age and ISAR scores.

**Additional file 2.** Comparison of the success of stage 1, postoperative complications and device removal for different age cut-offs.

## Data Availability

All data generated or analysed during this study are included in this published article [and its supplementary information files]. The original data files [confidential patient data] and statistical analyses are available from the corresponding author upon reasonable request.

## Abbreviations

ASA: American Society of Anaesthesiologists risk classification
CCI: Charlson Comorbidity Index
ICS: International Continence Society
ISAR: Identification of Seniors at Risk
NSAIDs: Nonsteroidal anti-inflammatory drugs
OAB: Overactive bladder
SD: Standard deviation
SNM: Sacral neuromodulation
VAS: Visual analogue scale
