# Supplementary material for: Does age alone negatively predict the outcome of sacral neuromodulation? A single-centre retrospective observational study
Source: BMC Urol. 2020 May 14;20:55. doi: 10.1186/s12894-020-00621-6 (PMC7227205; doi:10.1186/s12894-020-00621-6)
Supplement: Supplementary file 1 — Additional file 1. Comparison of age and ISAR scores. [file 12894_2020_621_MOESM1_ESM.docx]

**Additional file 1:**

Comparison of age and ISAR scores, presented as numbers.

|  | | **ISAR score** | | | | Total | P-value |
| --- | --- | --- | --- | --- | --- | --- | --- |
|  |  | 0 | 1 | 2 | 3 |  |  |
| **Age** | <70 years | 39 | 28 | 6 | 2 | 75 | Lack  of items. |
|  | ≥70 years | 6 | 10 | 2 | 2 | 20 |  |
| Total | | 45 | 38 | 8 | 4 | 95 |  |
