# Supplementary material for: Does age alone negatively predict the outcome of sacral neuromodulation? A single-centre retrospective observational study
Source: BMC Urol. 2020 May 14;20:55. doi: 10.1186/s12894-020-00621-6 (PMC7227205; doi:10.1186/s12894-020-00621-6)
Supplement: Supplementary file 2 — Additional file 2. Comparison of the success of stage 1, postoperative complications and device removal for different age cut-offs. [file 12894_2020_621_MOESM2_ESM.docx]

**Additional file 2:**

Comparison of the success of stage 1, postoperative complications and device removal for different age cut-offs, presented as numbers; *Chi-square test (α=0.05).

| Age cut-offs [years] |  | <70 | ≥70 | <65 | ≥65 | <60 | ≥60 | <55 | ≥55 | Total |
| --- | --- | --- | --- | --- | --- | --- | --- | --- | --- | --- |
| Successful stage 1 | No | 27 | 12 | 23 | 16 | 22 | 17 | 15 | 24 | 39 |
|  | Yes | 48 | 8 | 43 | 13 | 39 | 17 | 29 | 27 | 56 |
| Total | | 75 | 20 | 66 | 29 | 61 | 34 | 44 | 51 | 95 |
| P-value* | | 0.053 | | 0.064 | | 0.186 | | 0.200 | |  |
| Postoperative complications | No | 32 | 7 | 29 | 12 | 26 | 15 | 17 | 24 | 41 |
|  | Yes | 16 | 1 | 14 | 1 | 13 | 2 | 12 | 3 | 15 |
| Total | | 48 | 8 | 43 | 13 | 39 | 17 | 29 | 27 | 56 |
| P-value | | Lack of items. | | Lack of items. | | Lack of items. | | Lack of items. | |  |
| Device removal | No | 43 | 8 | 39 | 12 | 35 | 16 | 25 | 26 | 51 |
|  | Yes | 5 | 0 | 5 | 0 | 5 | 0 | 5 | 0 | 5 |
| Total | | 48 | 8 | 44 | 12 | 40 | 16 | 30 | 26 | 56 |
| P-value | | Lack of items. | | Lack of items. | | Lack of items. | | Lack of items. | |  |
